# Nelumbo genome database, an integrative resource for gene expression and variants of Nelumbo nucifera

**DOI:** 10.1038/s41597-021-00828-8

**Published:** 2021-01-29

**Authors:** Hui Li, Xingyu Yang, Yue Zhang, Zhiyan Gao, Yuting Liang, Jinming Chen, Tao Shi

**Affiliations:** 1 CAS Key Laboratory of Aquatic Botany and Watershed Ecology, Wuhan Botanical Garden, Chinese Academy of Sciences, Wuhan, 430074 China; 2 Center of Conservation Biology, Core Botanical Gardens, Chinese Academy of Sciences, Wuhan, 430074 China; 3 University of Chinese Academy of Sciences, Beijing, 100049 China; 4 Wuhan Institute of Landscape Architecture, Wuhan, 430081 China

**Keywords:** Plant molecular biology, Agricultural genetics

## Abstract

Sacred lotus (*Nelumbo nucifera*, or lotus) is one of the most widely grown aquatic plant species with important uses, such as in water gardening and in vegetable and herbal medicine. A public genomic database of lotus would facilitate studies of lotus and other aquatic plant species. Here, we constructed an integrative database: the Nelumbo Genome Database (NGD, http://nelumbo.biocloud.net). This database is a collection of the most updated lotus genome assembly and contains information on both gene expression in different tissues and coexpression networks. In the NGD, we also integrated genetic variants and key traits from our 62 newly sequenced lotus cultivars and 26 previously reported cultivars, which are valuable for lotus germplasm studies. As applications including BLAST, BLAT, Primer, Annotation Search, Variant and Trait Search are deployed, users can perform sequence analyses and gene searches via the NGD. Overall, the valuable genomic resources provided in the NGD will facilitate future studies on population genetics and molecular breeding of lotus.

## Abstract (summary)

**Table:** 

Measurement(s)	reference genome data • whole genome sequencing • transcriptome
Technology Type(s)	Hi-C • PacBio Sequel System • Illumina sequencing • RNA sequencing • DNA sequencing
Sample Characteristic - Organism	Nelumbo nucifera

Machine-accessible metadata file describing the reported data: 10.6084/m9.figshare.13487271

## Background & Summary

Sacred lotus (*Nelumbo nucifera*, or lotus) is an early-diverging eudicot with important value in terms of understanding the origin and evolution of eudicots^1,2^. Lotus has a widespread native distribution, ranging from Asia to northern Australia, and it is one of the most economically important aquatic plant species, with widespread uses, such as in water gardening and in vegetable and herbal medicine^3–5^. In horticulture, lotus is classified into three cultivated types according to their utilization: seed lotus, flower lotus and rhizome lotus. The genome of lotus (2n = 16, assembly size = 821.2 Mb) has been sequenced and assembled, providing an unprecedented opportunity for genetic studies and molecular breeding of lotus^6–8^.

**Fig. 1:**
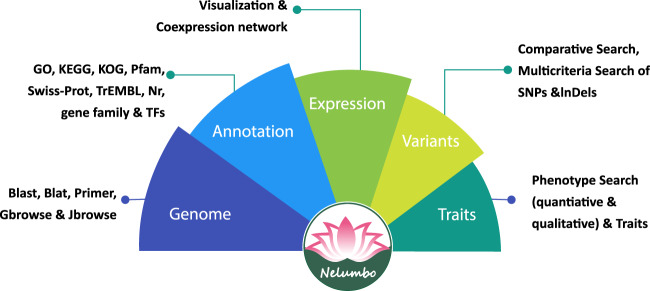
A schematic of the data collection and utilities for the Nelumbo Genome Database (NGD).

Since the first draft genome assembly of the lotus variety China Antique was released^9^, many genomic studies have been carried out on lotus, such as whole-genome resequencing^7,10,11^, transcriptomic^12–14^, miRNA-based^15–17^ and gene family studies^18,19^. The recent chromosome-level assembly of the China Antique genome facilitates genome-wide studies of functional genes and the evolution of lotus, but a web-based public database of lotus is still unavailable^8^. Due to public demand for an integrative genomic resource of lotus, we report our Nelumbo Genome Database (NGD, nelumbo.biocloud.net), which comprehensively houses, processes and integrates the newest assembly of lotus variety China Antique, the expression profiles of various tissues, genetic variants and phenotypes of 88 key lotus cultivars **(**Fig. 1**)**.

### Major datasets

The NGD is a collection of the new lotus assembly and annotations^8^, of which 34,481 genes harbor complete ORFs (> = 30 aa). A total of 28,676 genes were defined as high-confidence genes, with 14,991, 20,878, 15,276, 5,924, 29,095, 20,325, and 28,310 annotated genes in the KOG, PFAM, GO, KEGG, Nr, SwissProt and TrEMBL databases, respectively **(**Table 1**)**. The NGD also houses the sequences of 150,589 unique transcript isoforms based on RNA-seq and PacBio SMRT methods from our previous studies^14,17^. It also contains the sequences of 1,517 lotus transcription factors (TFs), which are classified into 56 TF (sub)families. Furthermore, sequence data of lotus gene families, transposable elements (TEs) and other repeats are also present in the NGD, and information concerning gene expression levels and highly coexpressed genes from data from a coexpression (WGCNA) network based on 69 RNA-seq samples from 11 lotus tissues (seed coat, cotyledon, receptacle, carpel, stamen, petal, rhizome, leaf, root, petiole and apical bud tissues) is present in the NGD. Furthermore, information concerning a total of 26,939,834 high-quality SNPs (single nucleotide polymorphisms), 4,177,974 InDels and key horticultural traits from 88 lotus cultivars is present in the NGD.

**Table 1:** Summary of functional annotations of lotus genes in different databases.

Database	Number of annotated genes
GO	15276
KEGG	5924
KOG	14991
Pfam	20878
Swiss Prot	20325
TrEMBL	28310
Nr	29095

### Uses

Through data collection and downstream processing, our platform provides the most complete lotus genome assembly for browsing via GBrowse or JBrowse^20,21^. Genes, DNA sequences, amino acids, SNPs and InDels can be viewed via GBrowse. The gene information page includes gene-splicing structures, sequences, and functional annotations such as those from the PFAM, KEGG, GO, KOG, SwissProt, TrEMBL and Nr databases. RNA-seq-based expression profiles across different tissues are also retrievable and can be visualized via a heatmap. Searching genes by keywords is also possible in the NGD. Additionally, coexpressed genes of a query gene in the WGCNA-derived network can be retrieved by setting a weight threshold; these coexpressed genes are likely involved in the same biological process as the query gene. Coding sequences and genomic sequences can be searched based on sequence similarity via BLAST or BLAT. Primers for the PCR experiments can also be designed directly in the NGD.

## Methods

### Data processing

Gene predictions on our chromosome-level genome assembly were performed using transcriptomes, gene homology and *ab initio* identification. A list of publicly available RNA-seq datasets, which mainly contain samples of the China Antique variety, were downloaded from the NCBI SRA database (Online-only Table 1**)**. First, the corrected consensus PacBio full-length transcripts were mapped to the lotus reference genome using GMAP^14^. RNA-seq reads (Illumina) were then mapped to the genome using the HISAT2-StringTie pipeline^22^. All the transcripts were further merged using TACO^23^. Coding DNA sequences (CDSs) of transcripts were predicted using Transdecoder (https://github.com/TransDecoder). Second, homology-based gene prediction was performed using GeMoMa, which used genome sequences and gene coordinates from *Arabidopsis thaliana*^24^, *Carica papaya*^25^, *Vitis vinifera*^26^, *Macadamia ternifolia* (Proteales)^27^ and *Brachypodium distachyon*^28^ as inputs. Third, *ab initio* prediction was performed using Braker2, which used transcript coordinates of RNA-seq as a guide^29^. Finally, all predictions were merged, and for each gene with more than one gene model (transcripts), the longest one was chosen as the representative gene model. Genes with an ORF less than 30 aa were discarded. Further, high-confidence gene sets were defined as those whose genes that either were homologous to those in other plant species in Plant Plaza 4.0 (https://bioinformatics.psb.ugent.be/plaza/versions/plaza_v4_dicots/) or were supported by RNA-seq (FPKM > 0.1). To quantify the expression of each gene in different lotus tissue samples, FPKM values across different RNA-seq samples were obtained via StringTie^22^. A coexpression network of different genes based on the expression profile was constructed using the WGCNA (v1.0) package in R^30^. Specifically, genes with an average FPKM > 0.1 and a coefficient of variation (CV) of gene expression (FPKM) > 2 were retained for the WGCNA. Genes were clustered hierarchically based on Topological Overlap Matrix^31^ and were assigned to nine modules (minimum module size of 600 and minimum module similarity of 0.5). The weight values between genes were used to represent the connectivity between genes.

Gene functions were annotated using the Gene Ontology (GO), KEGG, KOG, Pfam, SwissProt, TrEMBL, and Nr databases via KOBAS 3.0, BlastKOALA, PfamScan and BLAST^32,33^. As protein domains are conserved units shared by related genes, we clustered genes into domain families (gene families) according to the HMM Pfam domain annotations^34^. In addition, all transcription factors (TFs) were predicted and clustered into TF families via PlantTFDB 4.0^35^.

There were 88 recorded lotus cultivars chosen in this study as representing various floral traits (color, shape, flowering time, etc.). Among these cultivars, 62 were sequenced in our current study, while the sequences of 26 with detailed phenotypic records were downloaded from the NCBI database **(**Online-only Table 2**)**. Genomic DNA was first extracted from young leaves by the CTAB method^36^, and then DNA libraries were constructed by cutting the DNA into 250~280 bp fragments using a NEBNext® Ultra DNA Library Prep Kit for Illumina (NEB, USA) following the manufacturer’s recommendations. Paired-end reads (PE150) were sequenced on an Illumina NovaSeq. 6000 (San Diego, CA, USA), which generated approximately 16 × -depth data for each cultivar sample. Clean reads were obtained by removing the adapters and low-quality reads, including those comprising > 10% N, with < 20% low-quality bases, with low-quality/ambiguous fragments at the read ends within a 5 bp window and with a quality < 20 via FASTX-Toolkit (http://hannonlab.cshl.edu/fastx_toolkit/). The clean reads were mapped to the reference genome by BWA-men^37^. Variants were subsequently called by pipeline via GATK 4.0 (Genome Analysis Toolkit) with further SNP hard-filtering parameters (“QD < 2.0 || FS > 60.0 || MQ < 40.0 || MQRankSum < -12.5 || ReadPosRankSum < -8.0” and InDel hard-filtering parameters of “QD < 2.0 || FS > 200.0 || ReadPosRankSum < -20.0”)^38^. The phenotypes and some images of these cultivars were collected from reference books^39,40^; the phenotypes were further validated across two years of field investigations. Images of floral traits for these cultivars displayed in the NGD were taken mostly during flowering at the Wuhan Institute of Landscape Architecture (Wuhan, China).

### Database construction

All genomic sequence, annotation, expression, and genetic variation data were stored via MySQL on a Ubuntu server. A user-friendly website was developed using HTML5 and JavaScript; this website which can be accessed through different browsers, such as Google Chrome and Firefox. Gene models and transcript isoforms are provided via GBrowse and JBrowse. Heatmaps of gene expression are plotted via R, and query searches are achieved via JavaScript and Java. Common utilities for genomic studies such as BLAST, BLAT and Primer Design are also deployed and accessible.

## Data Records

The genomic raw PacBio sequencing data are available in the NCBI Sequence Read Archive (SRA) database under accession numbers SRR7549129^41^ and SRR7549130^42^, and the Illumina and Hi-C sequencing data are deposited under SRR7615553^43^ and SRR7631523^44^, which helped us in our genome assembly (Online-only Table 1**)**. Raw whole-genome resequencing reads for 62 strains can be downloaded from the NCBI database under Bioproject accession SRP173547^45^, and the resequencing data of the other 22 cultivars are also accessible via the NCBI SRA^46,47^ (Online-only Table 2). The latest assembly and annotations of the ‘China Antique’ lotus variety is deposited in the Nelumbo Genome Database (Download links: http://nelumbo.biocloud.net/downloadData/download?path = NNU.genomic.fa and http://nelumbo.biocloud.net/downloadData/download?path = NNU.gff3). Additionally, this Whole Genome Shotgun project has been deposited at DDBJ/ENA/GenBank under the accession DUZY00000000, and the version described in this paper is version DUZY01000000^48^. Improved gene and repeat (including transposable element) predictions (GFF3), coding and peptide sequences (FASTA), gene and transcript functional annotations, gene expression and coexpression profiles, SNP and InDel variations, phenotypic traits and images for 88 lotus strains have been uploaded into the Figshare database^49^ and deployed in the newly developed Nelumbo Genome Database (http://nelumbo.biocloud.net).

## Technical Validation

Quality control of genome annotation, expression, and genome resequencing was performed during data processing for the NGD.

### Genome annotation

We used a set of conserved single-copy plant genes from the BUSCO database to assess the completeness of gene annotations^50^. Compared with previous annotations of the lotus variety China Antique (BUSCOs = 74.6%), our new annotation version provides much improved, complete BUSCOs (97.5%), and 41,140 out of 46,713 annotated genes with either complete or partial ORFs (88%) were validated by 69 transcriptome datasets **(**Figure S1a**)**, which suggests relatively high quality and completeness of the genome assembly and gene annotations (Table 2**)**.

**Table 2:** BUSCO assessment of the completeness of gene annotation.

	Gene number^a^	BUSCO ratio^a^	Gene number^b^	BUSCO ratio^b^
**Complete BUSCOs**	1404	97.5%	1074	74.6%
**Complete and single-copy BUSCOs**	750	52.1%	947	65.8%
**Complete and duplicated BUSCOs**	654	45.4%	127	8.8%
**Fragmented BUSCOs**	16	1.1%	152	10.6%
**Missing BUSCOs**	20	1.4%	214	14.9%
**Total BUSCO groups searched**	1440		1440	

^a^The current genome assembly and annotation of var. China Antique^8^.

^b^Early genome assembly and annotation of var. China Antique^9^.

### Gene expression

**Fig. 2:**
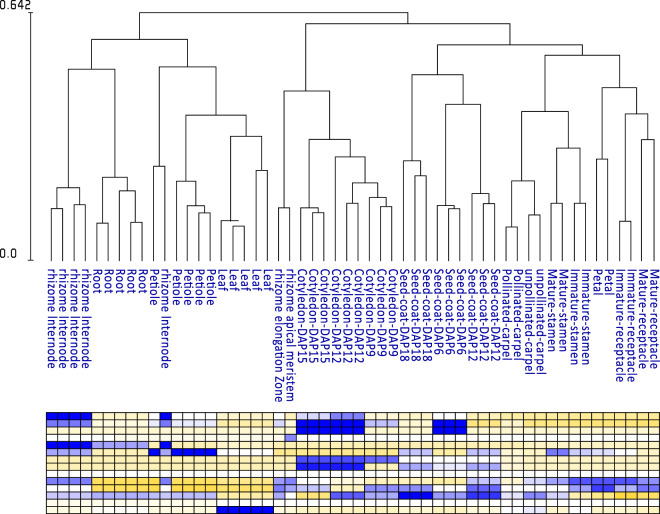
Hierarchical clustering of different RNA-seq samples based on a gene expression matrix (FPKM) from the lotus variety China Antique. Note that only a small portion of the genes are shown in the heatmap.

To ensure that the FPKMs of genes accurately reflect the gene expression in different tissues and at different developmental stages, hierarchical clustering of gene FPKMs across different RNA-seq samples of the variety China Antique was performed via Expander 6.0^51^. All sample repeats clustered together, while all developmental stages from the same tissue clustered together, except for one petiole sample, and the relative expression of randomly selected genes in different tissues was validated through qRT-PCR **(**Fig. 2 and Figure S1b**)**. Therefore, we confirmed the accuracy of FPKM as an indicator of lotus gene expression.

### Whole-genome resequencing

**Fig. 3:**
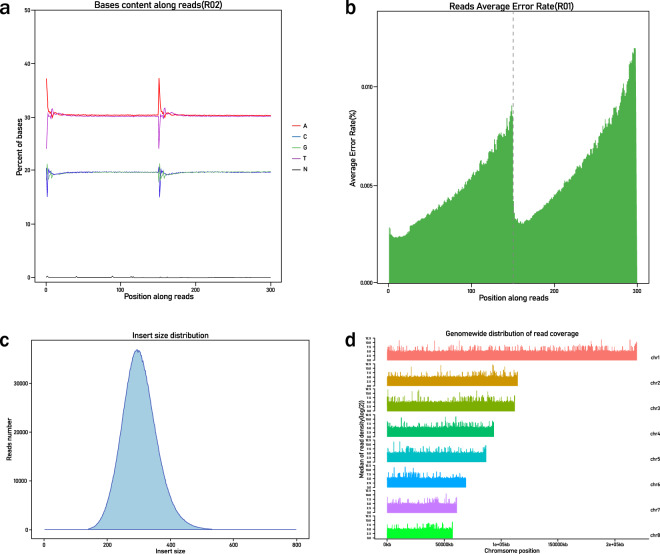
Quality evaluation of the genome resequencing data of cultivars, including the base content (**a**), error rate (**b**), insert size distribution (**c**) and log-transformed read coverage across eight lotus chromosomes (**d**), as demonstrated by the example of lotus cultivar Xiaoxia. The resequencing quality met the criterion for downstream variant calling analyses.

**Fig. 4:**
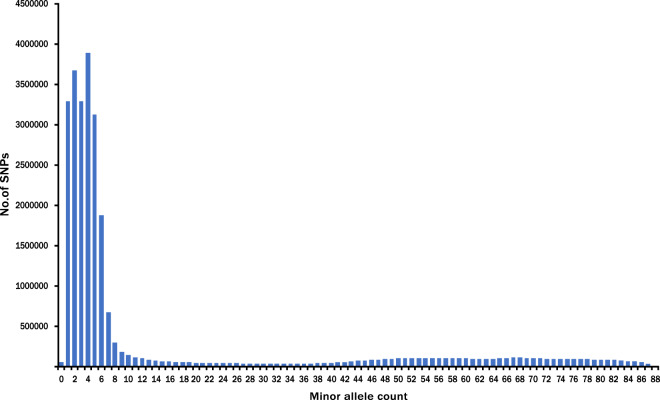
Distribution of SNPs according to the minor allele count (MAC) across 88 lotus cultivars.

Before genome mapping, adapters and low-quality Illumina reads were filtered and removed (see the Methods). Base content, error rate, insert size distribution and log-transformed read coverage across the lotus chromosomes were checked, all of which met the criteria for downstream analyses **(**Fig. 3**)**. The quality of genome mapping was also checked. The average mapping rate for cultivars from this study was 99.18%, while the numbers in the other cultivars collected from two previous studies were 98.98% and 99.13% **(**Online-only Table 2**)**. The average depth for the cultivars from the current study was 16.1×, while the depth was 12.4× and 11.8× for cultivars from the other two reports **(**Online-only Table 2**)**. To ensure the final quality of SNPs and InDels called by the GATK pipeline, stringent hard-filtering parameters were set (see the Methods). Because the majority of alleles in the SNP data set are expected to be shared by at least two individuals, we plotted the frequency of SNPs according to the minor allele count (MAC) across the 88 cultivars. Indeed, most of the SNPs had MACs ≥ 2, while the SNP density peaked around MACs of four or five **(**Fig. 4**)**. SNP variants were further validated and visualized using IGVtools (http://software.broadinstitute.org/software/igv/igvtools) **(**Figure S1c**)**.

## Supplementary information

Supplementary file

## Data Availability

The genomic and transcriptomic sequence data were produced by corresponding software provided by the sequencing platform manufacturer, and the software (including versions, parameters and settings) used for genome assembly was cited in the Methods section, with default parameters used when no detailed parameters were mentioned. The code for the NGD construction in Java is available in the Figshare database^49^.

## Online-only Tables

**Online-only Table 1:** Information and source of genomic data used in Nelumbo Genome Database.

Datatype	Platform	Cultivar Name	Utility	Tissue/developmental stage	Accession number (NCBI SRA)
DNA	PacBio SMRT	China Antique	Genome assembly	Young leaf	SRR7549129
DNA	PacBio SMRT	China Antique	Genome assembly	Young leaf	SRR7549130
DNA	Illumina & Hi-C	China Antique	Genome assembly	Young leaf	SRR7615553
DNA	Illumina & Hi-C	China Antique	Genome assembly	Young leaf	SRR7631523
RNA	PacBio SMRT	China Antique	Gene & isoform prediction	mixed tissue samples	SRR8182148
RNA	Illumina	China Antique	Gene expression	Seed-coat-DAP6	SRR7880135
RNA	Illumina	China Antique	Gene expression	Seed-coat-DAP6	SRR7880135
RNA	Illumina	China Antique	Gene expression	Seed-coat-DAP6	SRR7880135
RNA	Illumina	China Antique	Gene expression	Seed-coat-DAP12	SRR7880134
RNA	Illumina	China Antique	Gene expression	Seed-coat-DAP12	SRR7880134
RNA	Illumina	China Antique	Gene expression	Seed-coat-DAP12	SRR7880134
RNA	Illumina	China Antique	Gene expression	Seed-coat-DAP18	SRR7880133
RNA	Illumina	China Antique	Gene expression	Seed-coat-DAP18	SRR7880133
RNA	Illumina	China Antique	Gene expression	Seed-coat-DAP18	SRR7880133
RNA	Illumina	China Antique	Gene expression	Petal	SRR8169126
RNA	Illumina	China Antique	Gene expression	Petal	SRR8169126
RNA	Illumina	China Antique	Gene expression	Immature-stamen	SRR8169123
RNA	Illumina	China Antique	Gene expression	Immature-stamen	SRR8169123
RNA	Illumina	China Antique	Gene expression	unpollinated-carpel	SRR8169124
RNA	Illumina	China Antique	Gene expression	unpollinated-carpel	SRR8169124
RNA	Illumina	China Antique	Gene expression	Immature-receptacle	SRR8169125
RNA	Illumina	China Antique	Gene expression	Immature-receptacle	SRR8169125
RNA	Illumina	China Antique	Gene expression	Mature-stamen	SRR8169127
RNA	Illumina	China Antique	Gene expression	Mature-stamen	SRR8169127
RNA	Illumina	China Antique	Gene expression	Pollinated-carpel	SRR8169128
RNA	Illumina	China Antique	Gene expression	Pollinated-carpel	SRR8169128
RNA	Illumina	China Antique	Gene expression	Mature-receptacle	SRR8169129
RNA	Illumina	China Antique	Gene expression	Mature-receptacle	SRR8169129
RNA	Illumina	China Antique	Gene expression	Cotyledon-DAP9	SRR6432938
RNA	Illumina	China Antique	Gene expression	Cotyledon-DAP9	SRR6432938
RNA	Illumina	China Antique	Gene expression	Cotyledon-DAP9	SRR6432938
RNA	Illumina	China Antique	Gene expression	Cotyledon-DAP12	SRR6432941
RNA	Illumina	China Antique	Gene expression	Cotyledon-DAP12	SRR6432941
RNA	Illumina	China Antique	Gene expression	Cotyledon-DAP12	SRR6432941
RNA	Illumina	China Antique	Gene expression	Cotyledon-DAP15	SRR6432942
RNA	Illumina	China Antique	Gene expression	Cotyledon-DAP15	SRR6432942
RNA	Illumina	China Antique	Gene expression	Cotyledon-DAP15	SRR6432942
RNA	Illumina	China Antique	Gene expression	lotus root	SRR826691
RNA	Illumina	China Antique	Gene expression	Leaf	SRR830399
RNA	Illumina	China Antique	Gene expression	Leaf	SRR831069
RNA	Illumina	China Antique	Gene expression	Leaf	SRR831070
RNA	Illumina	China Antique	Gene expression	Leaf	SRR831071
RNA	Illumina	China Antique	Gene expression	Leaf	SRR831072
RNA	Illumina	China Antique	Gene expression	rhizome Internode	SRR831082
RNA	Illumina	China Antique	Gene expression	rhizome Internode	SRR831083
RNA	Illumina	China Antique	Gene expression	rhizome Internode	SRR831084
RNA	Illumina	China Antique	Gene expression	rhizome Internode	SRR831086
RNA	Illumina	China Antique	Gene expression	rhizome Internode	SRR831087
RNA	Illumina	China Antique	Gene expression	Petiole	SRR831088
RNA	Illumina	China Antique	Gene expression	Petiole	SRR831089
RNA	Illumina	China Antique	Gene expression	Petiole	SRR831090
RNA	Illumina	China Antique	Gene expression	Petiole	SRR831167
RNA	Illumina	China Antique	Gene expression	Petiole	SRR831168
RNA	Illumina	China Antique	Gene expression	Root	SRR831169
RNA	Illumina	China Antique	Gene expression	Root	SRR831170
RNA	Illumina	China Antique	Gene expression	Root	SRR831171
RNA	Illumina	China Antique	Gene expression	Root	SRR831172
RNA	Illumina	China Antique	Gene expression	Root	SRR831173
RNA	Illumina	China Antique	Gene expression	rhizome apical meristem	SRR831190
RNA	Illumina	China Antique	Gene expression	rhizome elongation Zone	SRR831693
RNA	Illumina	cultivated rhizome lotus (CRL) plants	Gene expression	Apical buds	SRR879365
RNA	Illumina	wild flower lotus (WFL) plants	Gene expression	Apical buds	SRR879367
RNA	Illumina	Zhouou	Gene expression	rhizome (stolon/internode)	SRR2052526
RNA	Illumina	Zhouou	Gene expression	rhizome (stolon/internode)	SRR2052539
RNA	Illumina	Zhouou	Gene expression	rhizome (later swelling stage/internode)	SRR2052541
RNA	Illumina	Fenhonglingxiao	Gene expression	rhizome (stolon/internode)	SRR2052538
RNA	Illumina	Fenhonglingxiao	Gene expression	rhizome (stolon/internode)	SRR2052542
RNA	Illumina	Zhouou	Gene expression	rhizome (later swelling stage/internode)	SRR2052551
RNA	Illumina	Zhouou	Gene expression	rhizome (middle swelling stage/internode)	SRR2052560
RNA	Illumina	Fenhonglingxiao	Gene expression	rhizome (middle swelling stage/internode)	SRR2052568
RNA	Illumina	Fenhonglingxiao	Gene expression	rhizome (middle swelling stage/internode)	SRR2052569
RNA	Illumina	Fenhonglingxiao	Gene expression	rhizome (later swelling stage/internode)	SRR2052570
RNA	Illumina	Zhouou	Gene expression	rhizome (later swelling stage/internode)	SRR2052571
RNA	Illumina	Fenhonglingxiao	Gene expression	rhizome (later swelling stage/internode)	SRR2052573
DNA	Illumina	shentong	Whole-genome resequencing	young leaf	SRR8325858
DNA	Illumina	fozuolian	Whole-genome resequencing	young leaf	SRR8325857
DNA	Illumina	yuloutai	Whole-genome resequencing	young leaf	SRR8325860
DNA	Illumina	jiaoyangzuiwu	Whole-genome resequencing	young leaf	SRR8325859
DNA	Illumina	xiaolianzuo	Whole-genome resequencing	young leaf	SRR8325862
DNA	Illumina	huoyan	Whole-genome resequencing	young leaf	SRR8325868
DNA	Illumina	yongshi	Whole-genome resequencing	young leaf	SRR8325864
DNA	Illumina	chongbanbayilian	Whole-genome resequencing	young leaf	SRR8325863
DNA	Illumina	ziyulian	Whole-genome resequencing	young leaf	SRR8325866
DNA	Illumina	hongwanlian	Whole-genome resequencing	young leaf	SRR8325865
DNA	Illumina	baixuegongzhu	Whole-genome resequencing	young leaf	SRR8325880
DNA	Illumina	baiwanwan	Whole-genome resequencing	young leaf	SRR8325879
DNA	Illumina	yudie	Whole-genome resequencing	young leaf	SRR8325878
DNA	Illumina	fenwanlian	Whole-genome resequencing	young leaf	SRR8325877
DNA	Illumina	taizhenchuyu	Whole-genome resequencing	young leaf	SRR8325884
DNA	Illumina	manaohong	Whole-genome resequencing	young leaf	SRR8325883
DNA	Illumina	luoxiayingxue	Whole-genome resequencing	young leaf	SRR8325882
DNA	Illumina	mudanxianzi	Whole-genome resequencing	young leaf	SRR8325881
DNA	Illumina	pinghuqiuyue	Whole-genome resequencing	young leaf	SRR8325872
DNA	Illumina	tianshanbitai	Whole-genome resequencing	young leaf	SRR8325871
DNA	Illumina	lihuabai	Whole-genome resequencing	young leaf	SRR8325836
DNA	Illumina	hongjuan	Whole-genome resequencing	young leaf	SRR8325837
DNA	Illumina	xinlingmei	Whole-genome resequencing	young leaf	SRR8325834
DNA	Illumina	shuangjie	Whole-genome resequencing	young leaf	SRR8325835
DNA	Illumina	fenghuazhengmao	Whole-genome resequencing	young leaf	SRR8325840
DNA	Illumina	juhong	Whole-genome resequencing	young leaf	SRR8325841
DNA	Illumina	cenglinjinran	Whole-genome resequencing	young leaf	SRR8325838
DNA	Illumina	fenmudan	Whole-genome resequencing	young leaf	SRR8325839
DNA	Illumina	chanjuan	Whole-genome resequencing	young leaf	SRR8325843
DNA	Illumina	cuitai	Whole-genome resequencing	young leaf	SRR8325844
DNA	Illumina	zixiabei	Whole-genome resequencing	young leaf	SRR8325870
DNA	Illumina	bixuedanxin	Whole-genome resequencing	young leaf	SRR8325869
DNA	Illumina	xiaofengliangyue	Whole-genome resequencing	young leaf	SRR8325853
DNA	Illumina	xiaotaihong	Whole-genome resequencing	young leaf	SRR8325847
DNA	Illumina	nanjinghong	Whole-genome resequencing	young leaf	SRR8325874
DNA	Illumina	shuimeiren	Whole-genome resequencing	young leaf	SRR8325873
DNA	Illumina	yubanbai	Whole-genome resequencing	young leaf	SRR8325876
DNA	Illumina	yuhua	Whole-genome resequencing	young leaf	SRR8325875
DNA	Illumina	liuhuo	Whole-genome resequencing	young leaf	SRR8325867
DNA	Illumina	xiaoxia	Whole-genome resequencing	young leaf	SRR8325861
DNA	Illumina	baiyun	Whole-genome resequencing	young leaf	SRR8325895
DNA	Illumina	baiqianye	Whole-genome resequencing	young leaf	SRR8325848
DNA	Illumina	jinzhuluoyupan	Whole-genome resequencing	young leaf	SRR8325849
DNA	Illumina	hanhong	Whole-genome resequencing	young leaf	SRR8325850
DNA	Illumina	fengximudan	Whole-genome resequencing	young leaf	SRR8325851
DNA	Illumina	suzhibingzi	Whole-genome resequencing	young leaf	SRR8325852
DNA	Illumina	chongbanyizhangqing	Whole-genome resequencing	young leaf	SRR8325842
DNA	Illumina	zhongshanhongtai	Whole-genome resequencing	young leaf	SRR8325854
DNA	Illumina	shennvzi	Whole-genome resequencing	young leaf	SRR8325855
DNA	Illumina	yanzhilu	Whole-genome resequencing	young leaf	SRR8325856
DNA	Illumina	xinjie	Whole-genome resequencing	young leaf	SRR8325892
DNA	Illumina	chaitoufeng	Whole-genome resequencing	young leaf	SRR8325891
DNA	Illumina	caiyunfeidu	Whole-genome resequencing	young leaf	SRR8325890
DNA	Illumina	lianxia	Whole-genome resequencing	young leaf	SRR8325889
DNA	Illumina	pizhenhong	Whole-genome resequencing	young leaf	SRR8325888
DNA	Illumina	haoyue	Whole-genome resequencing	young leaf	SRR8325887
DNA	Illumina	jiaoniang	Whole-genome resequencing	young leaf	SRR8325886
DNA	Illumina	danxiuqiu	Whole-genome resequencing	young leaf	SRR8325885
DNA	Illumina	hongdenggaozhao	Whole-genome resequencing	young leaf	SRR8325894
DNA	Illumina	fenxia	Whole-genome resequencing	young leaf	SRR8325893
DNA	Illumina	zhaoyun	Whole-genome resequencing	young leaf	SRR8325845
DNA	Illumina	baimeilian	Whole-genome resequencing	young leaf	SRR8325846
DNA	Illumina	hongtailian	Whole-genome resequencing	young leaf	SRR7159815
DNA	Illumina	shuguangxiu	Whole-genome resequencing	young leaf	SRR7159817
DNA	Illumina	donghuxinhong	Whole-genome resequencing	young leaf	SRR7159818
DNA	Illumina	xiangbihe	Whole-genome resequencing	young leaf	SRR7159820
DNA	Illumina	fengjuanhongqi	Whole-genome resequencing	young leaf	SRR7159821
DNA	Illumina	xingkongmudan	Whole-genome resequencing	young leaf	SRR7159822
DNA	Illumina	honghehuan	Whole-genome resequencing	young leaf	SRR7159825
DNA	Illumina	jinlinghuodu	Whole-genome resequencing	young leaf	SRR7159826
DNA	Illumina	xiaoqu	Whole-genome resequencing	young leaf	SRR7159827
DNA	Illumina	xiaojingling	Whole-genome resequencing	young leaf	SRR7159828
DNA	Illumina	zuidongfeng	Whole-genome resequencing	young leaf	SRR7159829
DNA	Illumina	hongyun	Whole-genome resequencing	young leaf	SRR7159830
DNA	Illumina	jinlingzhixing	Whole-genome resequencing	young leaf	SRR7159831
DNA	Illumina	danyue	Whole-genome resequencing	young leaf	SRR7159832
DNA	Illumina	qinhuaibaiyu	Whole-genome resequencing	young leaf	SRR7159833
DNA	Illumina	baige	Whole-genome resequencing	young leaf	SRR4308656
DNA	Illumina	baiyangdianhonglian	Whole-genome resequencing	young leaf	SRR4308659
DNA	Illumina	donghonghua	Whole-genome resequencing	young leaf	SRR4308651
DNA	Illumina	fenhonglingxiao	Whole-genome resequencing	young leaf	SRR4308650
DNA	Illumina	heilongjianghonglian	Whole-genome resequencing	young leaf	SRR4308657
DNA	Illumina	jianxuan17	Whole-genome resequencing	young leaf	SRR4308643
DNA	Illumina	puzheheibailian	Whole-genome resequencing	young leaf	SRR4308658
DNA	Illumina	qianbanlian	Whole-genome resequencing	young leaf	SRR4308655
DNA	Illumina	weishanhonglian	Whole-genome resequencing	young leaf	SRR4308645
DNA	Illumina	xihuhonglian	Whole-genome resequencing	young leaf	SRR4308660
DNA	Illumina	zhigaowushang	Whole-genome resequencing	young leaf	SRR4308649

**Online-only Table 2:** Mapping rate and depth on reference genome for 88 lotus cultivars.

Sample ID	Sample Name	Mapping rate	Depth	NCBI SRR
63	shentong	99.01%	11.9566	SRR8325858
74	fozuolian	98.97%	12.4726	SRR8325857
75	yuloutai	98.89%	14.0595	SRR8325860
86	jiaoyangzuiwu	98.12%	13.0646	SRR8325859
102	xiaolianzuo	99.00%	13.4425	SRR8325862
107	huoyan	98.81%	12.3096	SRR8325868
116	yongshi	98.96%	11.8835	SRR8325864
146	chongbanbayilian	99.13%	11.7565	SRR8325863
160	ziyulian	99.07%	13.6243	SRR8325866
163	hongwanlian	98.68%	13.5703	SRR8325865
169	baixuegongzhu	99.07%	12.748	SRR8325880
218	baiwanwan	99.24%	16.2777	SRR8325879
221	yudie	98.71%	16.6949	SRR8325878
235	fenwanlian	99.32%	16.2768	SRR8325877
239	taizhenchuyu	99.28%	16.1965	SRR8325884
274	manaohong	99.38%	16.5347	SRR8325883
296	luoxiayingxue	99.40%	16.9159	SRR8325882
305	mudanxianzi	99.23%	16.5897	SRR8325881
317	pinghuqiuyue	99.40%	18.1952	SRR8325872
319	tianshanbitai	99.35%	17.3546	SRR8325871
322	lihuabai	99.30%	18.5732	SRR8325836
334	hongjuan	99.09%	17.2609	SRR8325837
338	xinlingmei	99.32%	16.7434	SRR8325834
365	shuangjie	99.06%	11.9673	SRR8325835
368	fenghuazhengmao	99.40%	18.83	SRR8325840
380	juhong	99.37%	17.0724	SRR8325841
381	cenglinjinran	99.29%	16.1654	SRR8325838
388	fenmudan	99.27%	15.0731	SRR8325839
419	chanjuan	99.34%	17.5767	SRR8325843
421	cuitai	99.36%	18.721	SRR8325844
440	zixiabei	99.29%	15.9078	SRR8325870
450	bixuedanxin	99.35%	17.1246	SRR8325869
451	xiaofengliangyue	99.41%	17.1499	SRR8325853
452	xiaotaihong	99.38%	17.3033	SRR8325847
453	nanjinghong	99.34%	16.9085	SRR8325874
461	shuimeiren	99.37%	16.9447	SRR8325873
467	yubanbai	99.30%	15.5537	SRR8325876
468	yuhua	99.35%	17.4529	SRR8325875
495	liuhuo	99.20%	15.6028	SRR8325867
566	xiaoxia	99.40%	17.5277	SRR8325861
591	baiyun	99.45%	16.3501	SRR8325895
600	baiqianye	99.32%	17.2098	SRR8325848
619	jinzhuluoyupan	99.43%	15.6951	SRR8325849
640	hanhong	97.50%	17.2591	SRR8325850
642	fengximudan	99.34%	17.7888	SRR8325851
643	suzhibingzi	99.48%	17.5635	SRR8325852
652	chongbanyizhangqing	99.50%	16.64	SRR8325842
653	zhongshanhongtai	99.40%	16.5431	SRR8325854
656	shennvzi	99.31%	16.3544	SRR8325855
674	yanzhilu	99.46%	17.481	SRR8325856
678	xinjie	97.81%	16.2709	SRR8325892
709	chaitoufeng	99.33%	15.3606	SRR8325891
770	caiyunfeidu	99.43%	16.9894	SRR8325890
782	lianxia	99.33%	18.4933	SRR8325889
807	pizhenhong	99.23%	16.1976	SRR8325888
832	haoyue	98.68%	16.1062	SRR8325887
846	jiaoniang	99.34%	16.6935	SRR8325886
847	danxiuqiu	99.37%	17.3691	SRR8325885
855	hongdenggaozhao	99.18%	15.4943	SRR8325894
896	fenxia	99.46%	15.3978	SRR8325893
908	zhaoyun	99.43%	16.3684	SRR8325845
911	baimeilian	99.45%	20.3636	SRR8325846
7159815	hongtailian	99.31%	11.5062	SRR7159815
7159817	shuguangxiu	99.18%	12.0447	SRR7159817
7159818	donghuxinhong	99.34%	11.7097	SRR7159818
7159820	xiangbihe	99.03%	11.582	SRR7159820
7159821	fengjuanhongqi	99.14%	11.1591	SRR7159821
7159822	xingkongmudan	98.93%	12.5915	SRR7159822
7159825	honghehuan	98.30%	11.5102	SRR7159825
7159826	jinlinghuodu	98.91%	14.5597	SRR7159826
7159827	xiaoqu	99.16%	12.7112	SRR7159827
7159828	xiaojingling	97.92%	11.7524	SRR7159828
7159829	zuidongfeng	99.33%	13.1111	SRR7159829
7159830	hongyun	99.24%	13.9575	SRR7159830
7159831	jinlingzhixing	99.27%	13.6104	SRR7159831
7159832	danyue	99.16%	12.4043	SRR7159832
7159833	qinhuaibaiyu	98.41%	11.6404	SRR7159833
BG	baige	99.36%	20.3992	SRR4308656
BYDHL	baiyangdianhonglian	99.11%	3.68911	SRR4308659
DH2	donghonghua	99.27%	22.267	SRR4308651
FH	fenhonglingxiao	99.04%	18.6101	SRR4308650
HLJHL	heilongjianghonglian	98.92%	3.93594	SRR4308657
JX	jianxuan17	99.27%	20.6081	SRR4308643
PZHBL	puzheheibailian	99.17%	5.14056	SRR4308658
QBL	qianbanlian	99.11%	3.83764	SRR4308655
WSHL	weishanhonglian	99.14%	7.54628	SRR4308645
XHHL	xihuhonglian	99.01%	4.30606	SRR4308660
ZGWS	zhigaowushang	99.07%	20.4065	SRR4308649
