# Supplementary material for: Nelumbo genome database, an integrative resource for gene expression and variants of Nelumbo nucifera
Source: Sci Data. 2021 Jan 29;8:38. doi: 10.1038/s41597-021-00828-8 (PMC7846841; doi:10.1038/s41597-021-00828-8)
Supplement: Supplementary file 1 — Supplementary file [file 41597_2021_828_MOESM1_ESM.docx]

**Supplementary** **Figure**

Figure S1. (a) The proportions of only-annotated coding genes without (I) and with (II) isoseq/RNA-seq assembled genes. (b) The qRT-PCR validation of four genes in lotus anther, leaf, petal, petiole and unpollinated carpel. (c) Visualization of SNP variants of lotus cultivars by IGVtools.
